# Can Digit Ratio and Gender Identity Predict Preferences for Consumption Options With a Distinct Gender Image?

**DOI:** 10.3389/fpsyg.2022.923709

**Published:** 2022-06-09

**Authors:** Tobias Otterbring, Christian T. Elbæk, Chaoren Lu

**Affiliations:** ^1^Department of Management, School of Business and Law, University of Agder, Kristiansand, Norway; ^2^Institute of Retail Economics, Stockholm, Sweden; ^3^Department of Management, Aarhus University, Aarhus, Denmark; ^4^Kunming Academy of Governance, Kunming, China

**Keywords:** digit ratio, prenatal testosterone, masculinity, femininity, gender identity, gendered marketing

## Abstract

This study investigated whether individuals’ preferences for masculine (vs. feminine) consumption options could be predicted by a biological sex cue (the 2D:4D digit ratio; a biomarker linked to prenatal testosterone exposure), and a psychological gender cue (self-perceived gender identity). Chinese participants (*N* = 216) indicated their preferences for a series of binary options that differed in their perceived gender image (e.g., romantic comedy vs. action thriller; pop music vs. hard rock), with one of the options evaluated as relatively more feminine and the other viewed as comparably more masculine. Participants also self-reported their gender identity and the length of their index and ring fingers, which was used to calculate their 2D:4D digit ratios. A low (male-typical) digit ratio and a masculine gender identity were both associated with more masculine preferences, regardless of participants’ biological sex. However, a low digit ratio predicted preferences for masculine consumption options only in female participants with a masculine gender identity, but not in those with a feminine gender identity. These findings add to the literature on whether and when biological sex cues and psychological gender cues can predict preferences for options with a distinct gender image and suggest that the connection between these cues is more complex in women than in men.

## Introduction

One biological sex cue presumed to reflect prenatal testosterone exposure, the 2D:4D digit ratio (i.e., the ratio between the length of the index and ring finger), has been discussed in connection to several facets of consumer behavior (e.g., Aspara and Van Den Bergh, 2014; Otterbring et al., 2018; Hand, 2020). Higher levels of prenatal testosterone exposure, as indexed by lower digit ratios, have been shown to correlate positively with aggression (Bailey and Hurd, 2005; Hönekopp and Watson, 2011), and increased preferences for risk taking (Brañas-Garza et al., 2018) as well as the development of personality characteristics associated with sensation seeking (Fink et al., 2006). Regarding consumer behavior, there is evidence that individuals with low (male-typical) digit ratios exhibit more positive attitudes toward high-status goods than those with high (female-typical) digit ratios (Wu et al., 2017) and show increased interest in such prestigious products when primed with status goals (Cornelissen and Palacios-Fenech, 2016).

Some scholars have argued that self-perceived gender identity (i.e., the degree to which an individual associates his or her self-concept with masculine or feminine characteristics) may have better explanatory power than biological sex cues^1^ in predicting consumer preferences (Fischer and Arnold, 1994; Palan et al., 2001). For example, people with a masculine gender identity, regardless of their biological sex (cf. Torgrimson and Minson, 2005), tend to evaluate products with a masculine (vs. feminine) gender image more favorably (Neale et al., 2016). Moreover, men with such a “manly” gender identity are more focused on gift-giving with a clear object focus (vs. person focus), suggesting that they prioritize gifts that can convey social status (Palan et al., 2001; see also Palan, 2001) or, alternatively, that they prefer things-oriented rather than people-oriented gifts (cf. Su et al., 2009; Lippa, 2010). These findings emphasize self-perceived gender identity as another potentially influential factor that may also be associated with people’s product preferences and consumption responses (Gupta and Gentry, 2016).

The main objective of the current research was to *formally test* the explanatory power of the aforementioned cues (i.e., individuals’ digit ratio and gender identity) in predicting preferences for masculine (vs. feminine) consumption options; that is, options that people perceive to convey a distinct gender image. In a consumption context, an option’s gender image can be thought of as being associated with the sex of its most likely user (Debevec and Iyer, 1986; Kacen, 2000; Palan, 2001). We also sought to *explore* whether digit ratio and gender identity may interact to predict preferences for options with a certain gender image. Below, we outline the theoretical arguments that justify our two primary predictions, while simultaneously providing a brief rationale for our exploratory test of interactive effects.

### Digit Ratio and Consumer Preferences

Higher prenatal exposure to testosterone and hence a lower digit ratio tends to be observed more often in men, while the opposite holds true for women (Fink et al., 2003). Nevertheless, despite meta-analytic results indicating a mean sex difference in the 2D:4D measure commonly classified as a small-to-moderate effect size (Hönekopp and Watson, 2010), there is large variation in this measure, meaning that numerous women have a male-typical digit ratio and that multiple men have a female-typical digit ratio. In general, male-typical digit ratios are positively correlated with traits and behaviors that can be conceived as masculine (e.g., aggression, need for power, competitive orientation, and risk taking), while female-typical digit ratios represent more feminine features (Manning, 2002; Fisher et al., 2010). Male-typical digit ratios are also associated with more favorable attitudes toward high-status goods (Wu et al., 2017), consistent with the more overarching observation that testosterone is linked to a stronger striving for status (Archer, 2006; Josephs et al., 2006; Ronay and Galinsky, 2011; Nave et al., 2018). Because status has historically been more strongly linked to masculine characteristics (Vandello et al., 2008; Cheng et al., 2010; Koenig et al., 2011; Weaver et al., 2013), and given the multitude of studies in which male-typical digit ratios have been shown to predict masculine traits, interests, and preferences (e.g., Fisher et al., 2010; Aspara and Van Den Bergh, 2014; Manning et al., 2017), we anticipate that individuals with male-typical digit ratios – regardless of their biological sex – will exhibit a greater preference for consumption options that can be perceived as masculine. Hence, we predict:

**P1:** A male-typical digit ratio is positively associated with people preferring consumption options with a more masculine gender image.

### Gender Identity and Consumer Preferences

The relationship between self-perceived gender identity and preferences for consumption options with a distinct gender image should arguably mirror that between digit ratios and said preferences (**P1**), such that people with a masculine gender identity – regardless of their biological sex – should be more prone to prefer options with a masculine rather than feminine gender image (Palan, 2001; Fugate and Phillips, 2010; Neale et al., 2016; Pohlmann and Chen, 2020). We base this assumption on ample academic work in marketing and consumer behavior, which has documented that people’s preferences tend to be congruent with their own gender identity (Fry, 1971; Worth et al., 1992; Fischer and Arnold, 1994; Feiereisen et al., 2009; Grohmann, 2009). Thus, we predict:

**P2**: A masculine gender identity is positively associated with people preferring consumption options with a more masculine gender image.

### Exploring Interactive Within-Sex Effects

Although we expect both individuals’ digit ratio and gender identity to be associated with their preferences for consumption options with a distinct gender image, regardless of their biological sex (as per **P1–P2**), there may still be within-sex variation in the relative strength of these factors. Indeed, aspects associated with gender and sexuality have previously been discussed as more strongly linked to biological factors in men (such as digit ratio), whereas social, psychological, and cultural factors (such as gender identity) have been assumed to carry a greater weight in women (Baumeister, 2000; Peplau, 2001; Lippa, 2003), although many female aspects of gender and sexuality still have a strong biological basis (Luoto et al., 2019). Yet, given the limited research addressing interactive within-sex effects between individuals’ digit ratio and their gender identity, we did not deem it adequate to create a formal prediction, but rather set out to explore whether such effects could be established.

## Methodology

### Participants and Statistical Power

A sample of 216 Chinese participants (*M*_*age*_ = 28 years, *SD* = 8.5; 50% female) took part in the study, which was conducted online. Participants aged 18 and above were eligible for the study, with nearly 70% of participants being 18–30 years old, and with the oldest participant aged 55 years. The sole reliance on participants of Chinese ethnicity should be interpreted in light of previous research, which has demonstrated digit ratios to differ significantly between ethnic groups (Millet and Buehler, 2018). Hence, by including a sample of exclusively Chinese participants, we achieved a more robust digit ratio measure. Given our one-tailed predictions (**P1–P2**), our sample size has a statistical power greater than 85% to detect effect sizes as small as *d* = 0.40, assuming a conventional alpha level of α = 0.05 (Cohen, 2013). Considering that the meta-analytic sex difference in digit ratios has an estimated effect size of roughly *d* = 0.40 (Hönekopp and Watson, 2010), our study is highly powered to detect this typical sex difference and, by extension, effect sizes of similar magnitude for our primary predictions.

### Procedure and Measures

Participants initially indicated their preferences on a series of 10 binary items, of which five were related to food and were included for the purpose of a different project (cf. Otterbring et al., 2021b). Of relevance for the current study, the five remaining items included consumption options unrelated to food that were assumed to differ in terms of their perceived gender image (romantic comedy vs. action thriller; environmentally friendly car vs. sports car; movie tickets vs. money; pop music vs. hard rock; *t*-shirt/top with light or warm colors vs. *t*-shirt/top with dark or cold colors)^2^. These items were adapted from previous related research (Moss et al., 2006; Nepomuceno et al., 2010; Aspara and Van Den Bergh, 2014).

Participants indicated their preferences on the items using a 7-point scale (1 = definitely alternative A; 7 = definitely alternative B) and their responses were averaged to form an index variable, with higher values representing preferences for options with a more masculine gender image (Cronbach’s α = 0.66). For the calculation of 2D:4D digit ratios, participants were asked to self-report the length of their index and ring fingers for both hands. Specifically, participants were asked to use a ruler to measure their finger lengths in mm (from the fingertip to the most proximal crease), with instructions similar to those used in previous research relying on self-reported, directly measured digit ratios (e.g., Manning et al., 2017; for an alternative self-report approach, see Buser, 2012). Comparing our results with those of the most similar previous investigation, which also used directly measured self-reported right hand digit ratios (Manning and Fink, 2008; *N* = 153,429), revealed no meaningful differences either for men (*d* = 0.02) or women (*d* = 0.01). Hence, our approach is arguably comparable to studies that have relied on similar measurement strategies in the past. Beyond these measures, participants indicated their height in cm, weight in kg, and replied to the 14-item horizontal and vertical individualism and collectivism scale (Sivadas et al., 2008). This scale was discarded due to low reliability estimates (αs ≤ 0.63) across three of its four dimensions.

**FIGURE 1:**
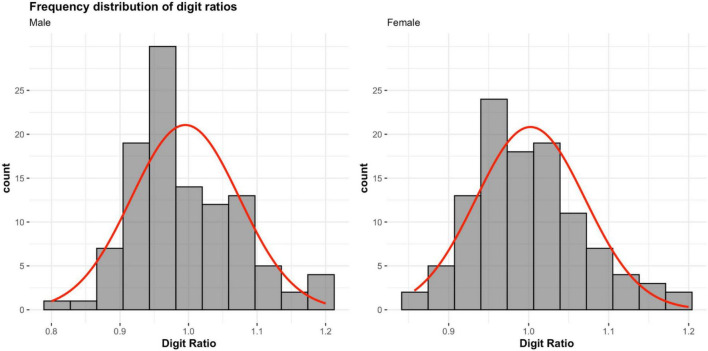
Frequency distributions of digit ratios between male (*N* = 108) and female participants (*N* = 108). Red lines indicate overall distribution of self-reported digit-ratios.

In the digit ratio analysis, we used participants’ right-hand digit ratio (*M*_2D:4D right_ = 0.999, *SD* = 0.074) following conventions in the digit ratio literature (cf. Apicella et al., 2015; Neyse et al., 2021). Digit ratios did not differ significantly between male (*M*_2D:4D right_ = 0.995, *SD* = 0.079) and female participants [*M*_2D:4D right_ = 1.002, *SD* = 0.068; *t*(214) = 0.69, *p* = 0.49, *d* = 0.10]. However, given that the means and standard deviations in our digit ratio measures are comparable with those of other published studies (e.g., Bull and Benson, 2006; Hand, 2020; Neyse et al., 2021), our approach of instructing participants to measure and self-report their finger lengths seems to have generated reasonable reliability. Moreover, as the sex-specific distributions of digit ratios reveal (see Figure 1), the frequency of male participants with a relatively more male-typical digit ratio (i.e., below 1; Hermann, 2017) was visibly greater. Indeed, a Pearson’s chi-square analysis using 2 (participant sex: male vs. female) × 2 (digit ratio: <1 vs. ≥1) crosstabs, with our digit ratio categories mirroring those used in previous research (Manning et al., 1998; Klimek et al., 2014; Kuna and Galbarczyk, 2018), revealed a larger proportion of male participants with a male-typical digit ratio (58.33%), and a larger proportion of female participants with a female-typical digit ratio (53.70%), χ^2^(1, *N* = 216) = 3.14, *p* = 0.05 (one-tailed), *d* = 0.24. Thus, although this effect size only represents a small effect according to current conventions in psychological science (Gignac and Szodorai, 2016; Funder and Ozer, 2019; Otterbring and Folwarczny, 2022), our digit ratio measure had some predictive validity, despite the absence of a significant sex difference in mean values.

To measure hunger, participants replied to six hunger items (e.g., “I need to do something about my hunger”) from Otterbring (2019), which were rated on a 7-point Likert scale (1 = strongly disagree; 7 = strongly agree), and averaged into a hunger index (α = 0.80). Hunger was used as a control variable in the main analysis, considering that this visceral state has been shown to influence people’s impatience, desire to acquire resources, and willingness to take risks (Xu et al., 2015; Williams et al., 2016; Skrynka and Vincent, 2019; Orquin et al., 2020), with such aspects typically perceived as masculine (Koot et al., 2009; Symmonds et al., 2010; Weaver et al., 2013; Chen et al., 2019).

To capture the construct of self-perceived gender identity, participants replied to Bem’s Sex Role Inventory in its short form (Bem, 1981a). This instrument measures the extent to which an individual associates his or her self-concept with aspects that are typically perceived as either masculine (e.g., “dominant”) or feminine (e.g., “compassionate”). To this end, participants indicated whether a set of adjectives were descriptive of themselves (1 = never true; 7 = always true), with 10 of the adjectives representing feminine aspects and 10 representing masculine aspects. We computed a difference score by subtracting participants’ femininity scores from their masculinity scores (cf. Bem, 1981b; Csathó et al., 2003). Thus, a positive (negative) value on this measure represents a masculine (feminine) gender identity. Supporting the predictive validity of this measure, and as reported elsewhere (Otterbring et al., 2021b), an independent samples *t*-test revealed that male participants (*M* = –0.25, *SD* = 1.02) had a more masculine gender identity than their female counterparts (*M* = –0.49, *SD* = 1.10), *t*(214) = 1.68, *p* = 0.05 (one-tailed), *d* = 0.22. Moreover, dividing the gender identity score into one feminine category containing all negative values (indicative a feminine gender identity) and one masculine category containing all values from 0 and upward (indicative of a more masculine gender identity) revealed that the proportion of male and female participants differed across these categories, χ^2^(1, *N* = 216) = 3.64, *p* = 0.04 (one-tailed), *d* = 0.26. Thus, more male (female) participants were found in the masculine (feminine) gender identity category (masculine: 56.14% male; feminine: 56.86% female). As such, both our focal predictors (i.e., participants’ digit ratio and gender identity) were predictively valid.

### Validation Study

We conducted a validation study on an independent sample of Chinese participants (*N* = 45; *M*_*age*_ = 28 years; *SD* = 7.3) to ensure that the consumption options used in each binary item differed significantly on the desired femininity/masculinity dimension in the main study. Participants rated all pairs of items (i.e., romantic comedy and action thriller; environmentally friendly car and sports car; movie tickets and money; pop music and hard rock; *t*-shirt/top with light or warm colors; and *t*-shirt/top with dark or cold colors) on a 7-point scale (1 = feminine; 7 = masculine). For each pair, the item assumed to convey masculinity was rated as significantly more masculine than the item assumed to convey femininity (all *p*s < 0.05). Moreover, a paired-samples *t*-test revealed that an index of all masculine items (*M* = 4.85, *SD* = 0.74) was evaluated as significantly more masculine than a similar index of all feminine items [*M* = 3.83, *SD* = 0.92; *t*(44) = 4.96, *p* < 0.001, *d* = 1.22], thus indicating that the items were suitable for use in the main study.

## Results

To analyze the data, we conducted a regression-based multiplicative moderation analysis (PROCESS Model 3; Hayes, 2017). Participants’ digit ratio (continuous) served as our first predictor, participants’ gender identity (continuous) acted as the second predictor, participants’ sex (female = 0; male = 1) served as the third predictor, and their preferences for consumption options with a distinct gender image (continuous) acted as the outcome variable. This analysis also included all two-way and three-way interactions between these predictors. We included hunger (continuous) as a covariate, given its documented effects on consumer preferences (e.g., Xu et al., 2015; Otterbring, 2019; Skrynka and Vincent, 2019), but its inclusion/exclusion does not influence the nature or significance of our primary predictions^3^. We opted for this analysis to maximize statistical power for our two primary predictions, while simultaneously allowing for tests of interactive within-sex effects between participants’ digit ratio and gender identity. Consistent with recommendations (Jones, 1954; Cho and Abe, 2013; Lakens et al., 2018; Otterbring et al., 2021c), we used one-tailed tests whenever we had one-sided predictions.

**FIGURE 2:**
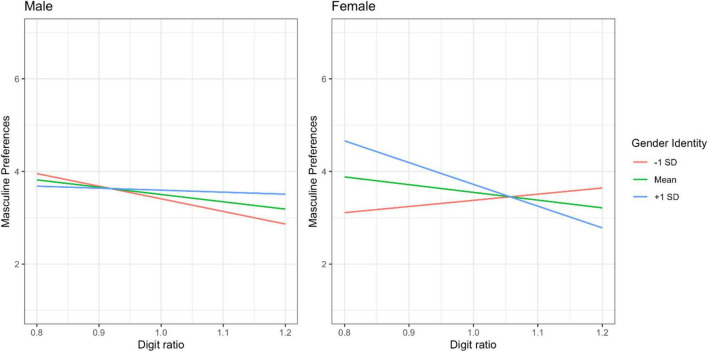
Male **(left)** and female **(right)** participants’ preferences for more masculine consumption options, depending on their digit ratio, which ranged from 0.80 to 1.20, and gender identity (*M* ± 1 *SD*), with *M* – 1 *SD* indicative of a feminine gender identity and *M* + 1 *SD* indicative of a masculine gender identity.

Our regression analysis, which both included participants’ digit ratio and their gender identity in the same model, explained roughly 10% of the variance in participants’ gender-imaged preferences and the overall model was statistically significant [*F*(8, 207) = 2.79, *p* = 0.006; *R*^2^ = 0.097]. Supporting **P1**, the link between participants’ digit ratios and their preferences for consumption options with a more masculine gender image was significant and negative (*b* = –2.03, *t* = –1.81, *p* = 0.04), such that participants with a male-typical digit ratio – regardless of their biological sex – preferred more masculine options. Moreover, in line with **P2**, the relationship between participants’ gender identity and their preferences for consumption options with a distinct gender image was significant and positive (*b* = 0.16, *t* = 2.03, *p* = 0.02), such that participants with a masculine gender identity – regardless of their biological sex – preferred more masculine options. Hunger as a covariate was also significantly associated with participants’ preferences (*b* = 0.25, *t* = 3.61, *p* < 0.001), such that hungry (vs. satiated) participants preferred options with a more masculine gender image. Participants’ biological sex was not significantly associated with gender-imaged preferences (*b* = –0.10, *t* = –0.59, *p* = 0.55), suggesting that participants’ digit ratio and gender identity had better predictive power in explaining such preference patterns. Interestingly, although all two-way interactions were non-significant (*p*s > 0.40), a statistically significant three-way interaction emerged (*b* = 4.35, *t* = 2.23, *p* = 0.03). To decompose this interaction, we tested the conditional digit ratio × gender identity interaction for male and female participants separately. For male participants, this interaction was non-significant (*p* = 0.28); however, it was significant for female participants (*p* = 0.05). As depicted in Figure 2, both digit ratio and gender identity seemed to independently predict male participants’ preferences for consumption options with a distinct gender image, such that a male-typical or masculine value on each of these metrics was associated with more masculine preferences. For female participants, however, this connection was more complex; a male-typical digit ratio was only associated with more masculine preferences for those female participants who simultaneously had a masculine – but not feminine – gender identity.

## Discussion

The results from the present study indicate that a biological sex cue (i.e., the 2D:4D digit ratio) as well as a psychological gender cue (i.e., self-perceived gender identity) can predict preferences for consumption options with a distinct gender image – regardless of an individual’s biological sex. Consistent with our predictions (**P1–P2**), we find that a low (male-typical) digit ratio and a masculine gender identity are both associated with an increased preference for more masculine consumption options, such as preferring an action thriller instead of a romantic comedy or favoring a sports car instead of an environmentally friendly car. However, this connection appears to be more complex for female participants, such that their digit ratio interacts with their gender identity to predict such preference patterns, with a male-typical digit ratio only linked to more masculine preferences among females with a masculine but not feminine gender identity. Yet, given the exploratory nature of these latter results, they should be interpreted with appropriate caution.

Our findings expand the literature on whether and when individuals’ digit ratio (e.g., Aspara and Van Den Bergh, 2014; Wu et al., 2017; Otterbring et al., 2018) and gender identity (e.g., Fry, 1971; Worth et al., 1992; Fischer and Arnold, 1994) may be associated with product purchases and preferences for alternatives with a distinct gender image. Considering the wide variety of alternatives available in our outcome measure, ranging from services and consumption experiences (music genres and movies) to cheap and expensive products (clothes and cars), our findings highlight the importance of considering both biological factors and psychological gender facets when designing promotional initiatives for products and services with a distinct gender image.

Apart from our main results, we also found that subjectively stated hunger was significantly associated with preferences for more masculine options. A possible explanation for this relationship could be that hungry individuals evaluate masculine options as carrying higher utility with respect to their status-signaling qualities (e.g., a sports car may be perceived as better able to signal status than an environmentally friendly car). Therefore, considering that resource scarcity, such as hunger, tends to activate a competitive mindset focused on resource acquisition (Xu et al., 2015; Goldsmith et al., 2018; Elbæk et al., 2022) and that status-signaling consumption is often used as a competition tactic (Otterbring et al., 2018; Nepomuceno and Stenstrom, 2021; Gasiorowska et al., 2022), it could be that hunger induces a stronger striving for status, thereby increasing consumers’ preferences for products and services with a salient masculine gender image.

## Limitations and Future Research

A potential limitation relates to our use of self-reported digit ratios. Although participants measured their finger lengths based on well-established guidelines, we acknowledge that this might have decreased the accuracy and reliability of our digit ratio measures to some extent (Caswell and Manning, 2009). In defense of this measurement approach, however, our obtained digit ratios were comparable to those reported in previous related research (Bull and Benson, 2006; Hand, 2020), including recent large-scale pre-registered investigations (Neyse et al., 2021). Moreover, our digit ratio measure exhibited predictive validity with respect to detecting sexual dimorphism in distributions, with more men (women) having a masculine (feminine) digit ratio. Nevertheless, future research should aim to assess the robustness of the current results with more objective digit ratio metrics, such as those obtained through hand scans, digital calibers, or direct measurements taken by trained professionals (Ribeiro et al., 2016).

The current study did not measure or control for participants’ sexual orientation. Because some studies have found within-sex differences in digit ratio and gender identity as a function of sexual orientation (e.g., Grimbos et al., 2010; Luoto et al., 2019), our results may be somewhat confounded by this factor (cf. Lippa, 2020).

Our work focused on self-reported preferences for consumption options with a distinct gender image. As such, it remains unclear whether the same results will emerge in more ecologically valid settings, such as in real retail stores. As scholars have argued that self-reported responses and artificial study settings may not always be indicative of consumer behavior “in the wild” (Cialdini, 2009; Gidlöf et al., 2021; Otterbring, 2021a,b; Saad, 2021), future research should try to replicate the present findings in actual field settings using naturalistic consumer choice or real sales data (Baumeister et al., 2007; Otterbring et al., 2020, 2021a; Rolschau et al., 2020).

Another direction for future research, despite that the digit ratio has been used extensively in the literature (Fink et al., 2003; Voracek and Loibl, 2009; Manning J. et al., 2014; Manning J. T. et al., 2014), is to rely on more objective tools to capture hormonal exposure, such as physiological measures of circulating testosterone or baseline levels on such hormonal factors (Nave et al., 2018; Dinsmore et al., 2021; Nepomuceno and Stenstrom, 2021). Although circulating testosterone in adults is not necessarily a reliable indicator of prenatal testosterone exposure, which is when sexual differentiation of the brain takes place (Arnold, 2020; McCarthy, 2020), future research should examine whether the current results can be extended to more objective measures of hormonal exposure. Additionally, while we used a monoethnic sample to circumvent large degrees of unaccounted variance in our digit ratio measures (Hönekopp and Watson, 2010; Millet and Buehler, 2018), future research should aim to test the robustness and generalizability of our findings in both WEIRD (Western, Educated, Industrialized, Rich, and Democratic) and non-WEIRD cultures (Henrich et al., 2010; Muthukrishna et al., 2020; Elbaek et al., 2021).

Finally, while not explicitly addressed in the current research, it is possible that consumers with a male-typical digit ratio or a masculine gender identity may be more motivated to buy products and services that are normally associated with femininity, such as sustainable, eco-friendly products (Brough et al., 2016), if such products are strategically promoted using masculine marketing messages and, when applicable, a corresponding “manly” packaging design. For example, the packaging of such products could consist of cues communicating a masculine gender image in terms of shapes (sharp and angular instead of round and curvy), textures (rough rather than glossy), and other verbal and visual elements conveying strength rather than gentleness (McNeill and Douglas, 2011; Steenis, 2019). Additionally, men with a male-typical digit ratio or a masculine gender identity may sometimes strategically choose products and services with a feminine connotation because favoring such choices could make them more desirable as mates under certain circumstances (Griskevicius et al., 2010; Borau et al., 2021; Palomo-Vélez et al., 2021). Future research should test these possibilities.

## Data Availability Statement

The raw data supporting the conclusions of this article will be made available by the authors, without undue reservation.

## Ethics Statement

This research was conducted without any funding source. Ethical review and approval were not required for the study on human participants in accordance with the local legislation and institutional requirements. Participants voluntarily and anonymously took part in the study. Written informed consent from the participants was not required to participate in this study in accordance with the national legislation and the institutional requirements.

## Author Contributions

TO developed the study design, analyzed the data, and coordinated the project. CL collected the data. TO and CE were responsible for the conceptualization. All authors drafted and revised the manuscript.

## Conflict of Interest

The authors declare that the research was conducted in the absence of any commercial or financial relationships that could be construed as a potential conflict of interest.

## Publisher’s Note

All claims expressed in this article are solely those of the authors and do not necessarily represent those of their affiliated organizations, or those of the publisher, the editors and the reviewers. Any product that may be evaluated in this article, or claim that may be made by its manufacturer, is not guaranteed or endorsed by the publisher.

## Footnotes

1 Admittedly, such claims can be thought of as representing a false dichotomy, considering that an individual’s psychological gender identity also has a biological basis (Arnold, 2017; Luoto et al., 2019).

2 While these binary choices may seem gender stereotypic, it should be noted that perceptions of femininity and masculinity are largely based on stereotypes (e.g., Briton and Hall, 1995; McCauley et al., 1988; Jussim et al., 2018), with most gender stereotypes in fact being more accurate than inaccurate at the aggregate level (e.g., McCauley et al., 1988; Swim, 1994; Jussim et al., 2018; Beeghly, 2021).

3 A reviewer requested us to also control for participants’ age (cf. Trivers et al., 2006). Adding age as a second covariate does not change the nature or significance of our results.
